# Autoantibody-dependent amplification of inflammation in SLE

**DOI:** 10.1038/s41419-020-02928-6

**Published:** 2020-09-09

**Authors:** Hantao Lou, Beata Wojciak-Stothard, Marieta M. Ruseva, H. Terence Cook, Peter Kelleher, Matthew C. Pickering, Juthathip Mongkolsapaya, Gavin R. Screaton, Xiao-Ning Xu

**Affiliations:** 1 Division of Immunology and Inflammation, Department of Medicine, Hammersmith Campus, Imperial College London, London, W12 0NN UK; 2 Department of Medicine, Centre for Immunology & Vaccinology, Chelsea and Westminster Hospital, Imperial College London, London, SW10 9NH UK; 3 Department of Medicine, Centre for Pharmacology and Therapeutics, Imperial College London, London, W12 0HS UK; 4 Division of Immunology and Inflammation, Centre for Complement and Inflammation Research, Imperial College London, London, W12 0NN UK; 5 Department of Infection and Immunity, Charing Cross Hospital North West London Pathology, London, W6 8RF UK; 6 Division of Medical Sciences, John Radcliffe Hospital, Oxford University, Oxford, OX3 9DU UK

**Keywords:** Cell death, Autoimmunity

## Abstract

Anti-double stranded DNA antibodies (anti-dsDNA) are a hallmark of SLE but their role in disease pathogenesis is not fully resolved. Anti-dsDNA in serum are highly heterogeneous therefore in this study, we aimed to dissect the functional specificities of anti-dsDNA using a panel of human monoclonal antibodies (humAbs) generated from patients with active lupus nephritis. A total of 46 ANA reactive humAbs were isolated and divided into four broad classes based on their reactivity to histones, DNA and *Crithidia*. Functional analysis indicated that one subclass of antibodies bound strongly to decondensed DNA areas in neutrophil extracellular traps (NETs) and protected NETs from nuclease digestion, similar to the sera from active SLE patients. In addition, these anti-dsDNA antibodies could stimulate type I interferon responses in mononuclear phagocytic cells, or NF-kB activity in endothelial cells, by uptake of NETs-anti-NETs immune complexes and subsequently trigging inflammatory responses in an Fc-gamma receptor (Fcg-R)-dependant manner. Together our data suggest that only a subset of anti-dsDNA antibodies is capable to amplify inflammatory responses by deposit in the nephritic kidney in vivo, protecting NETs digestion as well as uptake of NETs immune complexes into Fcg-R-expressing cells in vitro.

## Introduction

Systemic lupus erythematosus (SLE) is a multi-system autoimmune disease of varying severity, which commonly affects the skin and joints, but can also affect a variety of other organ systems. Most patients with SLE develop anti-nuclear antibodies (ANA) directed to a variety of nuclear components. Anti-dsDNA antibodies are highly specific, being found in 68–83% of SLE patients at some time during their illness^1–3^. Anti-dsDNA antibodies are associated with active SLE disease and deposit in the kidney of some lupus nephritis (LN) patients, which may contribute to the development of lupus nephritis (LN)^4–11^. There are several proposed mechanisms whereby anti-dsDNA can cause inflammation and contribute to disease pathogenesis, including the formation of immune complexes with chromatin exposed on apoptotic cells and the subsequent recruitment of Fcg-R myeloid cells or by the fixation of complement.

NETosis is a neutrophil-specific cell process (formation of NET occurs by two main pathways: a slow neutrophil-specific lytic cell death pathway and a more rapid non-lytic pathway from viable cells), which is characterized by the formation of NET in response to infectious (for example *C. albicans*) and sterile stimulation (for example anti-phospholipid antibodies, anti-RNP antibodies, IL-8 and monosodium uric acids)^12–14^. NETs are composed of decondensed chromatin and a range of neutrophil granular antimicrobial proteins including myeloperoxidase (MPO), neutrophil elastase (NE), LL37, etc. NETs have an important role during infection as they capture and neutralise pathogens^13,14^. Impairment in the clearance of apoptotic cells and NETosis are believed to be a potential source of autoantigens in SLE, which are proposed to trigger systemic autoimmune inflammation^10–12^. In this regard, it has been shown that activation of neutrophils by immune complexes containing nucleic acid can promote expression of pro-inflammatory cytokines, including type I interferon SLE^13–15^. Impairment of NETs degradation was observed in SLE patients and the undigested NETs was found to deposit in the kidney of some Lupus nephritis patients^10,16–18^. This suggests that impaired NETs digestion is associated with renal damage in SLE patients.

To gain more insight into the nature of the interaction between NETs and autoantibodies in lupus we set out to generate human monoclonal antibodies (humAb) from lupus patients that react with NETs. We generated a panel of 46 ANA reactive humAb and characterise their reactivity to dsDNA, histones and Crithidia luciliae antigen. A subset of five of these anti-ANA humAb was fully polyreactive, binding strongly to dsDNA, histone 1 and Crithidia. These five humAb also bind to NETs and inhibit NETs degradation. Furthermore, this subset of five humAb when in complex with NETs promote type I interferon expression upon uptake by blood monocytes and increases NF-kB expression in endothelial cells in an Fcg-R-dependent manner, providing a potential mechanism for autoantibody-driven amplification of inflammation.

## Materials and methods

### Patients, blood samples and donor cells

Blood samples were collected from two patients with active renal SLE for single plasmablast molecular cloning. Serum samples were also collected from patients undergoing diagnostic blood tests for suspected connective tissue disease and from SLE patients who were being monitored for active disease. The study was performed in accordance with the local Research Ethics Committee and Institutional approval. Sera from 65 patients with SLE were obtained from the Department of Infection and Immunity at North West London Pathology Trust Charing Cross Hospital. Ten ANA−ve patients were also recruited as negative controls. All SLE patients fulfilled the 1997 revised criteria of the American College of Rheumatology for the diagnosis of SLE. Clinical data on disease manifestations, treatments· renal function, ANA titre, complement and anti-dsDNA levels were collected from patient records. SLE disease activity was classified on the basis of symptoms, alteration in renal function and SLE treatment using the BILAG2004 criteria for constitutional, cutaneous, musculoskeletal and renal disease. Active disease was defined as the need to commence or increase cytotoxic, steroid and hydroxychloroquine therapy.

Peripheral blood mononuclear cells (PBMC) were isolated by Ficoll-Hypaque density-gradient centrifugation from healthy donors. Monocyte was isolated by CD14 magnetic beads while neutrophils were isolated by dextran sedimentation.

### Isolation of monoclonal antibodies from plasmablasts

Monoclonal antibodies were isolated from single plasmablasts. Briefly, plasmablasts (CD3-CD20^low^/-CD19^+^CD38^hi^CD27^hi^) were sorted by a FACS sorter (BD FACSARIA) into 96-well plates containing 10 µl RNase-inhibiting RT-PCR catch buffer (5 ml RNase-free water, 50 µl 1 M Tris pH 8 and 125 µl RNasin (Promega)). Plates were immediately sealed and frozen on dry ice and stored at −80 °C. Single-cell cDNA was synthesised in the original sort plates by adding 15 µl RT-PCR reaction mix. RT-PCR reaction mix contains 1 µl forward primer mix (1.2 µM), 1 µl reverse primer mix (1.2 µM) 1 µl dNTPs (200 µM), 5 µl 10× buffer, 0.5 µl PCR enzyme mix and 6.5 µl H_2_O. Individual IgH and IgL(k or λ) genes were amplified in the second round PCR reaction with HotstarTaq PCR kit. IgH and Ig λ genes are digested with *Age*I/*Sal*I or *Age*I/*Xho*I, respectively, prior to the ligation into the variable gene cloning site of the IgH/IgL expression vectors. The expression vectors are composed of the appropriate human constant region downstream of a murine immunoglobulin signal peptide and ampicillin-resistant gene. The Igk products were sequenced and amplified according to its gene family by another round of PCR. They were then digested with *Age*I/*BsiW*I before ligated into Igk expression vector. IgH and IgL chain-containing plasmids were mixed with PEI (Polyethylenimine) and transfected into human embryonic kidney fibroblast 293T cells. Cells were washed with DMEM/PBS 24 h after transfection and then cultured in protein-free media Supernatants are collected five-six days after transfection.

### Antibody purification

293T cells transfected with VH and VL plasmids were cultured in PF Ultradoma (Lonza) for 5 days before supernatant was collected, filtered (0.2 µm) and treated with MNase (2.5 U/ml) for 120 min. The supernatant is then loaded to a column packed with protein A beads and washed with PBS three times before eluted with glycine pH 2.7 and neutralised with Tris-HCl pH 8. The purified antibody is subsequently buffer-exchanged with PBS and stored in −80 °C.

### Determination of ANA, DsDNA and anti-histone antibodies

ANA-IIF screens on serum samples obtained from patients with suspected or know SLE was performed manually on Hep-2 cells (ANA-IIF) according to the manufacturer’s protocol (IMMCO Diagnostics, Ely, UK) and a titre of ≥1:160 was considered positive, leading to further antigen characterisation. ANA-IIF screen were performed on neat supernatant cultures of monoclonal antibodies isolated from SLE patients. Anti-DsDNA antibodies were detected by ELISA (ED-FDNA 100) according to the manufacturer’s instructions. Confirmation of positive DNA samples was performed using the Crithidia luciliae immunofluorescence (CLIF) test (BioSystems, Barcelona Spain). Anti-histone and anti-chromatin (ORGENTEC) ELISAs were performed according to the manufactures’ manual. Anti-histone/chromatin western blot protocol is described in the supplementary methods.

### Apoptotic cells binding

Jurkat cells were cultured in RPMI + 10% fetal calf serum (R10) and washed to be resuspended in RPMI at a concentration of 1 × 10^6^ cells/ml. Jurkat cells were then added to 6-well plate at 1 ml/well. For anti-Fas IgM killing, 100 ng/ml anti-Fas IgM was added to each well and the Jurkat cells were collected, washed after 60 min. The treated Jurkat cells were rested in R10 for 8 h. For UV killing, the plate was placed under UV (wavelength 320 nm) for 30 min followed by washing. The cells were cultured in R10 for 3 h. Apoptotic Jurkat cells were then co-incubated with the anti-dsDNA mAbs (5 µg/ml) for 1 h at 4 °C followed by FITC-conjugated anti-human IgG (Sigma), Annexin V and PE staining. The mAb binding to the apoptotic cells was analysed by flow cytometer and microscope.

### Generation and analysis of NETs binding and degradation

Neutrophils from healthy donors were isolated by dextran sedimentation according to standard protocols. Sterile 13-mm-round-glass coverslips were placed on 24-well plates and 5 × 10^5^ neutrophils were seeded on each well for 30 min at 37 °C. 500 ng/ml PMA or A23187 solution in RPMI was added to the wells to stimulate the neutrophils for 3 h. NETs were induced as described and co-incubated with either PBS or mAbs at various concentrations for 1 h and then washed with PBS. 1 U/ml Mnase or DNaseI was then added to the NET with 2 mM CaCl_2_ to kick start the digestion for 10 min at 37 °C. The supernatant was then transferred to a black plate and sytox orange added to the wells. The amount of digested DNA was then measured by fluorescence spectrometry. The number of NETs digested with 1 U/ml Mnase in PBS is defined as 100% digestion.

Neutrophil extracellular traps (NETs) were fixed with 4% paraformaldehyde and blocked with 5% goat serum. NET were then co-incubated with 10 µg/ml mAb followed by FITC-conjugated anti-human IgG. To visualise NETs, DAPI was added to the slides. Images were taken by fluorescent microscopy (OLYMPUS IX70).

**Fig. 1:**
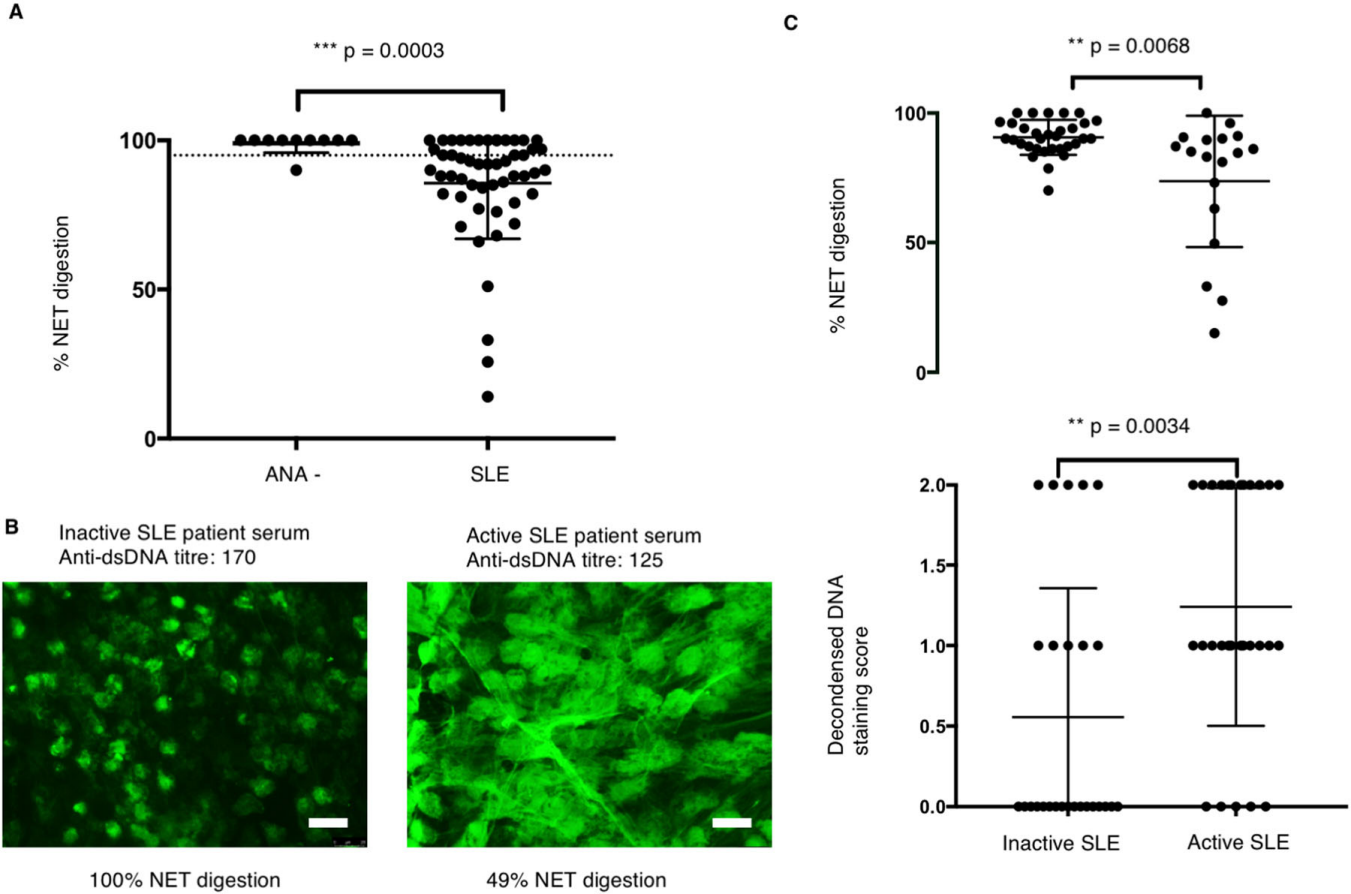
Serum from some active SLE patients protect NET from digestion. **a** NET digestion ability of ANA −ve donors (*n* = 10) and SLE patients (*n* = 51) were measured and compared. NET were stimulated by PMA followed by incubation with 10% serum for 6 h, and the amount of DNA in the supernatant was measured. 3 U/ml Mnase in PBS was used to digest NET, and the amount of digested NET is regarded as 100%. The percentage of NET digestion was calculated accordingly. Data represent mean of three repeats ± SEM, Mann–Whitney *U*. **b** NET binding of sera (10%) from 51 patients were studied by immunofluorescence microscopy. Representative images of inactive and active SLE serum (10% serum diluted in PBS) binding are illustrated (*n* = 51). The serum binding to NET was visualised by FITC-conjugated anti-human IgG antibodies. **c** NET digestion ability of active (*n* = 18) and inactive SLE patients (*n* = 33) was measured three times and compared. NET staining was scored for each SLE patients in a double-blind manner and analysed on a dot plot (*n* = 51). Data represent mean of three repeats, Mann–Whitney *U*.

During NETosis, chromatin becomes decondensed and relaxed to form long stretches. DNA-intercalating dye stains condensed DNA strongly but reacts weakly with decondensed DNA^19^. Serum binding to long stretches of decondensed DNA (denoted by arrows on Fig. 1) was visualised by anti-human IgG FITC. The decondensed DNA binding ability of serum (1:100) was scored double blindly by two immunologists at a grade of 0, 1 and 2^20,21^.

### Measurement of type I interferon secretion by monocytes

NETs were induced using the described protocol, washed with PBS and co-incubated with mAbs at the indicated concentrations for 1 h at 37 °C. NETs were then co-incubated with human monocytes isolated from healthy donors in RPMI-10 per cent FBS (fetal bovine serum). After 24 h, mRNA was isolated (RNeasy Plus Mini Kit, Qiagen) followed by cDNA synthesis (SuperScript IV, Thermofisher) and gene expression was measured by qRT-PCR (brilliant III SYBR Green, Agilent). Primers used *IFNA1* (forward: 5′-GGAGTTTGATGGCAACCAGT-3′, reverse: 5′-CTCTCCTCCTGCATCACACA-3′), *IFNB* (forward: 5′-AGCACTGGCTGGAATGAGAC-3′, reverse: 5′-TCCTTGGCCTTCAGGTAATG-3′).

### Endothelial cells NFκB luciferase reporter assay

HPAECs passaged between 3–6 passages were seeded into 96-well culture plates at 2 × 10^4^ cells/well. After reaching 90% confluence, HPAECs were infected with adenoviruses containing NFκB luciferase reporter construct (AdNFκB-luc)^4^ at multiplicity of infection (MOI) 1:100. 4 h post-infection, the medium was replaced with 50 μl of (ECGM-2) media, containing 0.15 µg/ml of NETs DNA with, or without control, dsDNA and LALA antibodies (100 µg/ml) Cells were then incubated for a further 24 h at 37 °C. NETs were pre-incubated with antibodies for 30 min at room temperature before addition to cells. Treatment with 10 ng/ml of TNF-α (Sigma) was used as a positive control for NF-κB activation. After 24 h incubation, cells were lysed with 1× lysis buffer (Promega) and 20 μl of each sample was transferred to white 96-well plates (Corning™) and combined with 100 μl of Luciferase Assay Reagent™ (Promega,). Luminescence, proportional to the level of NFκB-driven expression of luciferase, was measured in the Glomax™ luminometer.

### Accelerated nephrotoxic nephritis (NTN) mice model

C57BL/6 wild-type mice were purchased from Harlan Ltd. (Bicester, UK). All animal procedures were performed in accordance with institutional guidelines and under license by the UK government.

Accelerated NTN (ANTN) was induced by the i.v. injection of 200 μl of sheep nephrotoxic serum (a sheep Ig fraction containing anti-mouse GBM antibodies) into mice that had been sensitised with an intraperitoneal injection of 200 μg of sheep IgG (Sigma-Aldrich, Dorset, UK) in CFA (Sigma-Aldrich). Mice were housed in SPF conditions and individually ventilated cages.

### Kidney tissue staining

Anti-dsDNA monoclonal antibodies (200 μg/mice) were intraperitoneally into nephrotoxic nephritis (NTN) mice. After 6 h, the mice were killed and serum and kidney was harvested. Kidney samples were fixed with OCT and 2 µm sections were cut and stored at −80 °C. For staining, slides were soaked in acetone for 7 min, dried, washed with PBS and incubated with 5% goat serum (Sigma G9023-10 ml) for 1 h. Slides were dried and stained with anti-human/mouse IgG FITC diluted at 1:200 in blocking buffer (goat anti-human Fc-specific FITC, Sigma 100M4848 F5387) for 1 h at room temperature. Finally, slides were washed three times with PBS and mounted with the mounting media for visualisation under a microscope.

### Statistical analysis

All experiments were repeated at least in triplicate with four experimental repeats. Data are presented as means ± standard error deviation of the mean (SEM). All graphs and calculations were carried out with GraphPad Prism6 version 6.03 for Windows (GraphPad Software Inc). One-way ANOVA with Tukey multiple comparison post-test was used to determine statistical significance.

## Results

Several laboratories have reported that serum from some SLE patients can impair NETs digestion, implying the presence of a nuclease inhibitor and/or anti-NETs antibodies, which can either prevent access of endogeneous nucleases to or inhibit their activity on NETs. We measured NET nuclease-protection activity in polyclonal sera (10% neat serum is diluted in PBS) from a cohort of SLE patient (serum anti-DNA titre and NETs protection ability is provided in Supplementary Table 1). The patients were scored by BILAG into active and inactive SLE groups (as described in Methods). The ANA-ve control patients (confirmed by ANA staining and dsDNA ELISA test) were also enrolled as a negative control from Charing Cross Hospital.

### Active SLE patients show stronger binding to decondensed DNA of NETs

Fifty-one SLE patients sera were tested and ANA+ve SLE patient serum (10% serum diluted in PBS) displayed significantly lower NETs digestion than control ANA−ve serum in the presence of DNase (Fig. 1a). We then asked whether NETs protection is correlated with anti-NETs antibody binding by analysing the NETs digestion and NETs staining pattern of individual SLE and ANA− sera. None of the ANA-ve serum (10% diluted in PBS) bound to NETs while the majority of the SLE sera bound, indicating the presence of anti-NETs antibodies. Interestingly, sera from active SLE patients displayed significantly stronger binding to a decondensed area of NETs compared to inactive SLE serum at a similar level of anti-DNA antibody titre (Fig. 1b).

Next, we compared NETs protection between patients with active (*n* = 18) and inactive disease (*n* = 33). Serum (10% neat sera diluted in PBS) from active SLE patients showed significantly higher NETs protection than serum from inactive patients in the presence of DNase (Fig. 1c). However, we did not find a significant correlation between the anti-dsDNA level and NETs protection or NET binding (Supplementary Fig. 1). During NETs formation, chromatin decondensation occurs and NETs are mainly composed of these relaxed decondensed DNA^21,22^. We then quantified the decondensed DNA staining of SLE patients sera according to the methods (double-blinding scoring by two independent research scientists on a scale of 0–2). The decondensed DNA staining is also more predominant in active SLE patients (Fig. 1c).

### Anti-dsDNA antibodies are polyreactive

In order to dissect the heterogeneity of the polyclonal set of anti-NETs antibodies in the serum from SLE patients, we cloned 206 humAbs from single plasmablasts (CD3^−^, CD20^−^, CD19^+^, CD27^hi^, CD38^hi^) sorted from PBMC of two active SLE patients with renal disease (Table 1, Supplementary Fig. 2). Following heavy and light chain PCR amplification antibodies were expressed and screened for ANA activity.

**Fig. 2:**
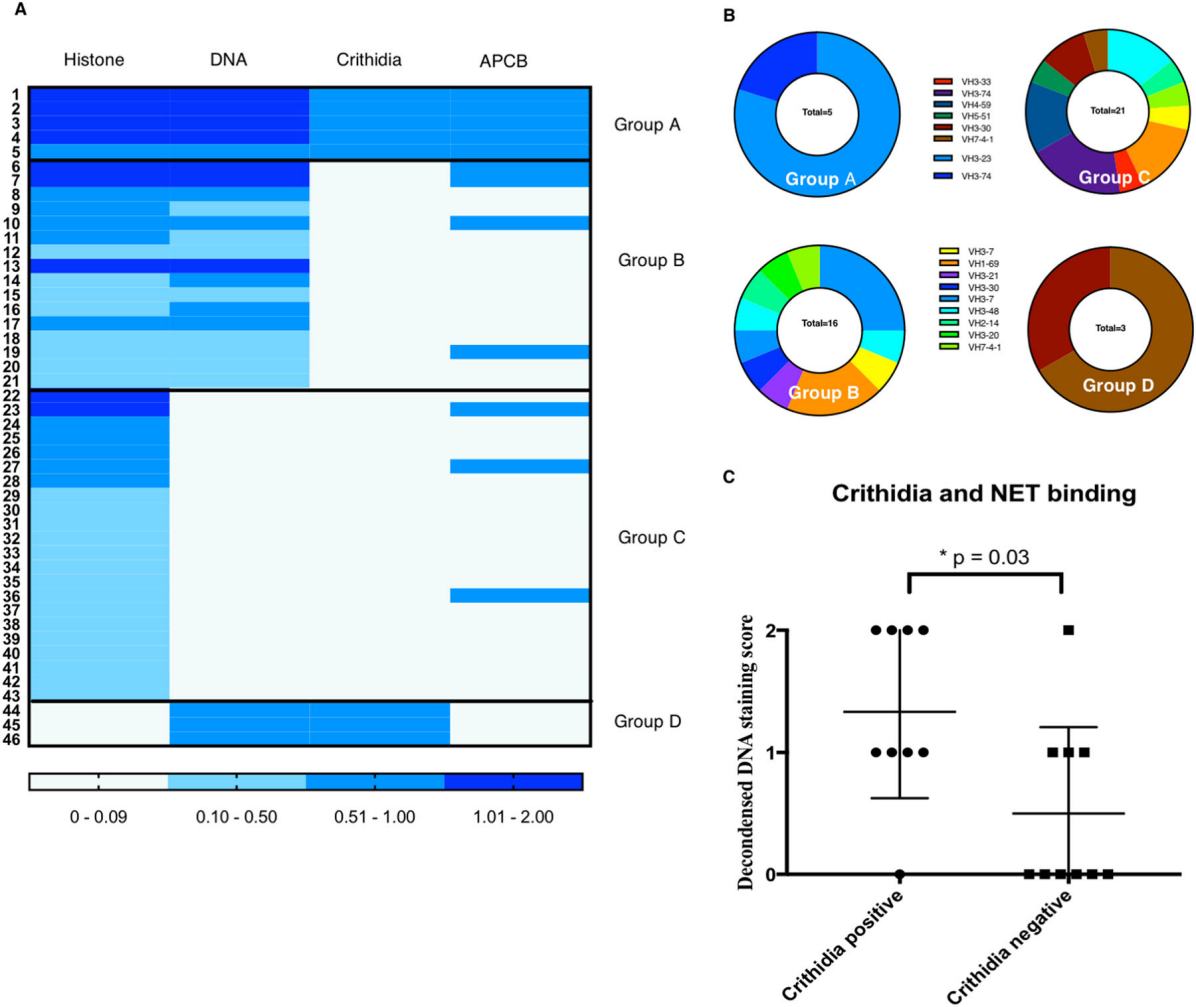
IgH gene features and reactivity of four groups of humAbs. **a** Heat-map summarises the mAbs (1 μg/ml) reactivity against human histones, naked DNA (DNA) and Crithidia kinetoplast and UV treated/FAS ligand-induced apoptotic cells (APCB). Antibodies are grouped according to their reactivity. APCB and Crithidia results were scored as either 0 (negative) or 1 (positive). ELISA was repeated for three times and mean was calculated for the table, *n* = 3. **b** Pie chart summarising antibody heavy chain gene family usage of each group of mAbs. Percentage of each gene family and the total number of mAbs were displayed. **c** The decondensed DNA binding score of the patients with positive crithidia staining or negative crithidia staining was compared. *N* = 18, Student *t*-test, *p* = 0.03.

In all, 206 humAbs were screened for ANA, using an indirect immunofluorescence assay (IFA) on fixed Hep-2 cell. Forty-six ANA positive ENA negative humAbs were isolated, and their reactivity against histones, naked DNA and Crithidia kinetoplasts determined. Many humAbs have been found to be cross-reactive with both histones and DNA, a feature commonly observed in SLE patients who produce a high frequency of polyreactive and self-reactive mature naive B cell^23,24^ (Fig. 2a). Furthermore, anti-dsDNA antibodies were shown to bind non-chromatin antigens including insulin, LPS, collagen, annexin II at high concentrations^25–27^. We assigned the 46 anti-ANA humAbs into four groups depending on their reactivity. Group A humAbs bound to all three antigens; histones, DNA and Crithidia and apoptotic cellls; group B bound to histones and DNA but not Crithidia, while group C bound to histones only and group D bound to DNA and Crithidia but not histones (Fig. 2a, b). Furthermore, 5/5 mAbs of group A bound to histone 1 while 2 group B mAbs bound to core histones (histone 2/3) (Supplementary Fig. 3). Interestingly, 4/5 of the group A humAbs use VH3-23 while groups B and C have a more diverse VH usage (Supplementary Fig. 4d). The heavy chain CDR3 region of group A humAbs are more positively charged than the other three groups. The length of CDR3, the number of mutations and light chain usage is similar between the four groups (Supplementary Fig. 4). Finally, we showed than monoclonal dsDNA antibodies, which bound to Crithidia (marker for moderate to higher affinity DsDNA antibodies compared to ELISA) had significantly increased DNA decondensed scores (marker for decondensed area of NETs binding) (Fig. 2c).

### Group A mAbs bind and protect NET from nuclease digestion

**Fig. 3:**
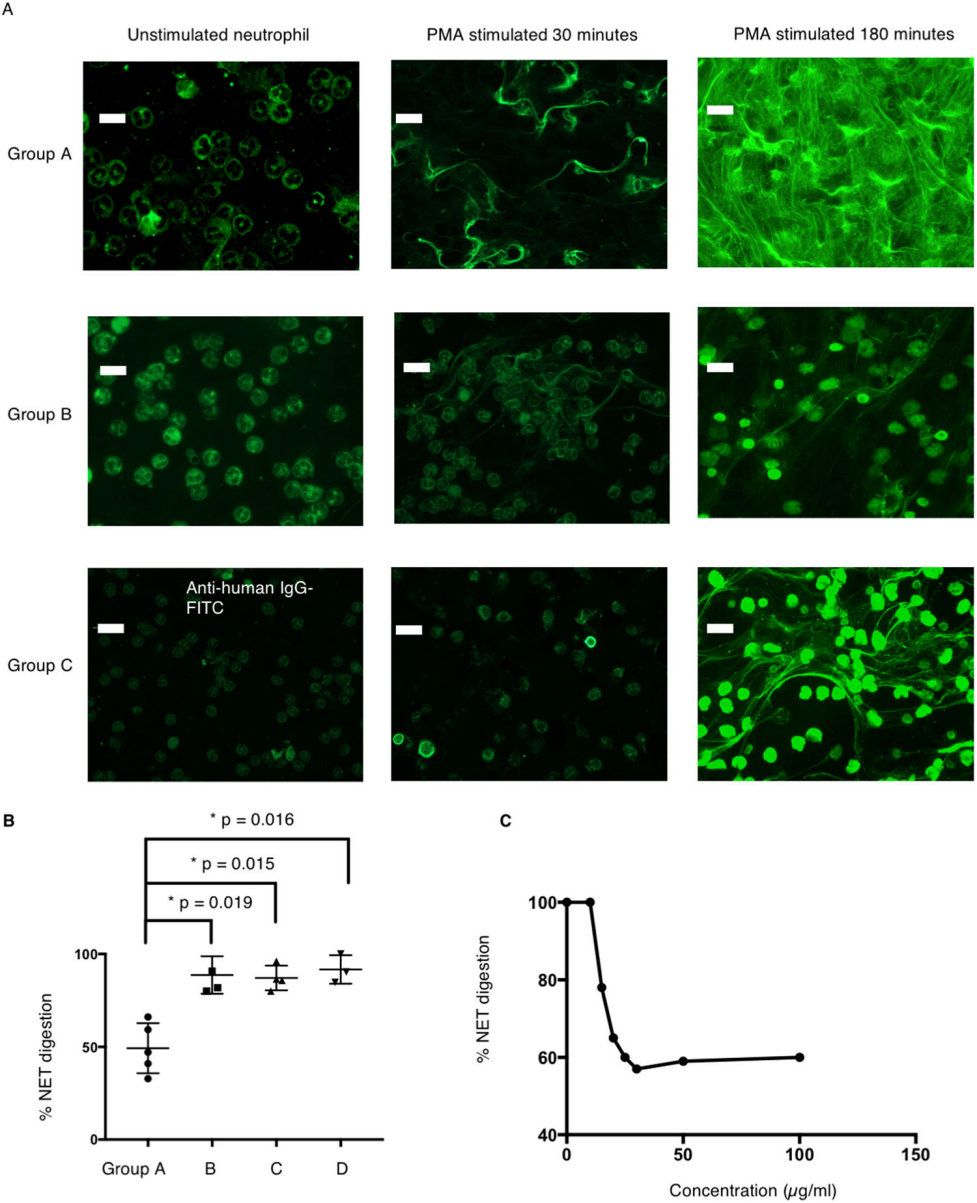
Binding of humAbs to neutrophils and NET. **a** Representative mAb binding to neutrophils and NET from group A, B and C. Group D mAbs. The antibody binding to NET was detected by FITC-conjugated anti-human IgG while NET was visualised by DAPI, bar length = 25 μm. Neutrophils were either unstimulated or stimulated with 100 ng/ml PMA for 30 or 180 min before staining. Groups A and B bound to neutrophil cytoplasm at 10 μg/ml as shown in the figure but did not bind to unstimulated neutrophil cytoplasm at 1 μg/ml. In total, 12/46 mAbs were positive for NET binding. **b** Percentage of NET digestion was measured in the presence of group A, B, C and D mAbs (*n* = 15) and displayed as a dot plot, repeated for three times. Data represent mean ± SEM, Error bars indicate mean with 95% CI and Mann–Whitney *U* was performed. **c** Group A mAbs protect NET from nuclease digestion in a dose-dependent manner, ranging from 1 to 100 μg/ml. The graph is representative of one group A mAb (557A3), *n* = 15.

Next, we examined the binding of humAbs to neutrophils and NET; 5/5 group A, 5/15 group B and 4/21 group C antibodies (10 μg/ml) bound to both unstimulated neutrophils and NETs, while none of the group D antibodies (10 μg/ml) bound neutrophils or NETs (Fig. 3a). The group A and B mAbs bind to NETs at 0.1μg/ml but did not bind to unstimulated neutrophil at 1 μg/ml (Supplementary Fig. 5B). Since the structure of NETs can vary with different stimuli, we examined the binding of monoclonal anti-dsDNA antibodies to NETs after stimulation with A23187 (divalent cation ionophore). Our results show a similar binding pattern to that observed with PMA stimulation (Supplementary Fig. 5A). However, the pattern of NETs staining differed between the humAbs from groups A, B & C. Group A showed strong binding to the decondensed DNA area of the NETs stimulated by either PMA or A23187 (Fig. 3a), similar to the staining pattern of sera from active SLE patients, which also showed strong NETs protection activity **(**Fig. 1b). Groups B and C antibodies showed predominant binding to the condensed DNA area of the NET (Fig. 3a), while group D antibodies did not stain NETs.

We were next interested to see if any of the NETs binding antibodies could protect NET from nuclease digestion. Neutrophils were stimulated with PMA or calcium ionophore A23187 for 3 h and NETs structure was imaged by staining with sytox orange. NETs were pre-incubated with humAbs prior to the addition of Micrococcal nuclease or DNaseI (1 U/ml MNase or DNase1 for 10 min at 37 °C) and the amount of digested DNA revealed by a fluorescent cytox orange assay. Group A antibodies (*n* = 5), which bound to decondensed DNA, showed strong NETs protection in a dose-dependent manner (from 1 to 100 μg/ml) (Fig. 3b, c), similar to 10% sera from active SLE patients (Fig. 1c). HumAbs targeting condensed DNA (*n* = 11), from groups B, C and D, failed to show significant inhibition (Fig. 3b).

### Group A mAbs enhance type I IFN expression and NF-kB expression

**Fig. 4:**
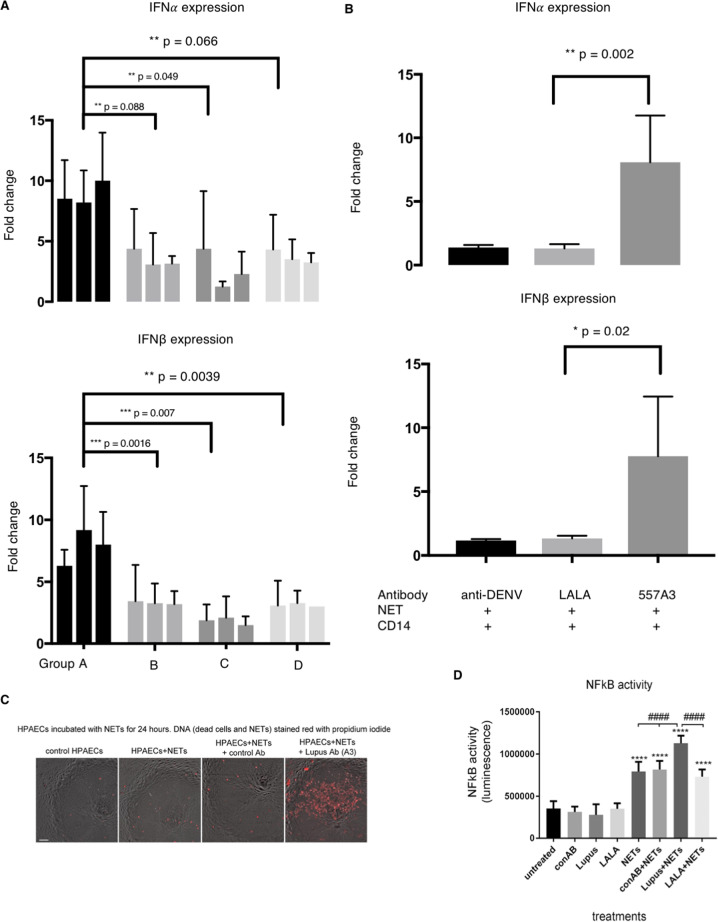
Group A antibodies enhance type I IFN and NF-kB. **a** Monocytes were co-incubated with NET and three humAbs from each group (*n* = 12). Type I IFN expression was then measured by qPCR and fold change were calculated against negative control (anti-DENV antibody). Error bars indicate mean of three repeats with 95% CI, two-way ANOVA. **b** Type I IFN mRNA levels are measured by incubating NET, monocyte and group A humAb-557A3 (dark grey) or 557A3-LALA (light grey). Error bars indicate mean with 95%CI Student *t*-test, *n* = 4. **c** Significantly more NET stayed on HPAECs after 24 h when co-incubated with group A humAb-557A3. HPAECs were incubated with NETs for 24 h in the presence of control and 557A3 before staining with propidium iodide. The addition of group A antibody markedly attenuated degradation of NETs (red, last image on the right). Bar = 50 µm. **d** HPAECs infected with AdNFkB-luc were incubated with NETs in the presence or absence of group A antibody 557A3. Following overnight incubation, NF-kB activity was measured in a luciferase reporter assay. ****P < 0.0001, comparisons with untreated control; ^####^*P* < 0.0001, comparisons, as indicated. ANOVA with Tukey post-test, *n* = 4.

We then studied the effects of anti-NET humAbs on primary human monocytes and endothelial cells to determine mechanistic links between the impairment of NETs digestion and inflammatory immune responses in SLE. Monocytes were co-incubated with NETs coated with 12 humAbs from groups A–D (3 from each group) and induction of type I IFN was determined by RT-PCR. Group A antibodies significantly enhanced type I IFN expression in monocytes compared to either the negative control antibody or the other 9 ANA mAbs (groups B, C and D) (Fig. 4a). Type I IFN enhancement is induced by group A mAbs (*n* = 3) in a dose-dependent manner (Supplementary Fig. 6A). We also tested the type I IFN expression induced by apoptotic cells co-incubated with 6 ApoC-binding humAbs from group A, B and C as an apoptotic cell is the other important source of extracellular DNA in addition to NETs. However, we did not observe significantly enhanced or suppressed type I IFN expression 24 h after the co-incubation (Supplementary Fig. 6B)

To further investigate the mechanism of the type I IFN enhancement by NETs immune complexes, we mutated the Fc portion of the heavy chain of group A mAb-557A3 by introducing the LALA mutation (mutations of leucine (L) to alanine (A) substitution at the position 234 and 235) known to restrict interaction with activating Fc-receptors. 557A3-LALA failed to enhance type I IFN when incubated with monocytes, suggesting that the process dependent on the uptake of antibody-NET complexes into the myeloid cells in an Fcg-R-dependent manner (Fig. 4b).

We then tested how anti-dsDNA antibody affects endothelial cells. Endothelial cells were coated with NET and humAbs for 24 h and amount of NET left on endothelial cells were visualised by DNA dye. NET-557A3 form stable immune complexes that stay on the endothelial cells after 24 h while NET alone or with group B antibodies were digested (Fig. 4c) It has been suggested that undigested NET may directly damage endothelial cells REF, so we next tested the ability of NET-humAb immune complexes to activate NF-κB in endothelial cells. NET-humAb immune complexes stimulated NF-κB activity in endothelial cells twofold compared to a control IgG1 anti-dengue humAb (Fig. 4d). HumAb-557A3-NET immune complex enhancement of NF-κΒ activity in endothelial cells was also Fcg-R dependent as the activity was lost when the LALA version of HumAb-557A3 was used (Fig. 4d).

### Group A mAbs deposit in nephritic kidney

**Fig. 5:**
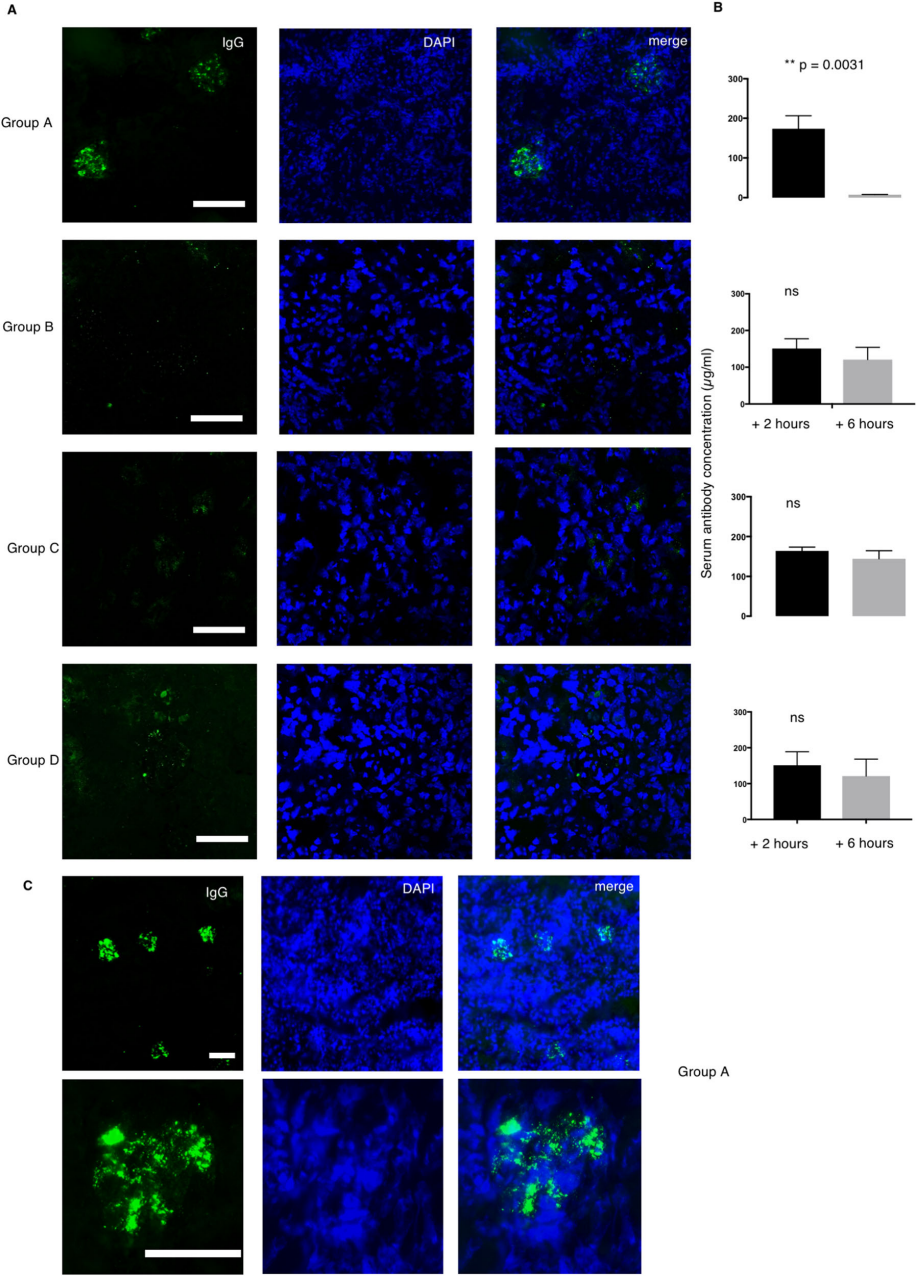
Group A antibody deposit in the kidney of nephritic mice. **a** Two humAbs from Group A, B, C and D (*n* = 8) were intraperitoneally injected to the nephritic mice. After six hours the mice were sacrificed to collect serum and kidney sample. IgG deposition was studied by staining the acetone fixed kidney with FITC-conjugated anti-human IgG (green) and DAPI (blue). **b** Serum human IgG level was measured at 0 and 6 h after humAb injection. Data represent mean ± SEM. Error bars indicate mean with 95% CI. *n* = 3. Student *t*-test. **c** The deposition was observed in multiple glomeruli seen at ×10 magnification and deposition pattern was visualised at ×40 magnification.

We were interested to determine whether the anti-NETs humAb would interact with inflamed tissue in vivo. We used an active autoimmune nephrotoxic nephritis (NTN) model that has predominant kidney inflammation driven by immune complexes deposition, mimicking lupus nephritis. To this end, we injected intraperitoneally 100 μg of representative humAbs from groups A–D (*n* = 8) into mice primed for immune complex-mediated nephritis (as described in “Materials and methods” section) at day 4. The concentration of circulating humAb was measured 2 and 6 h following injection. Group A humAb-557A3 and 157 B9 deposited exclusively in glomerular areas (Fig. 5a, c) and was completely cleared from the serum 6 h after injection (Fig. 5b). The groups B, C and D humAbs (*n* = 6) that do not protect NET did not deposit in the kidney and they remained in circulation after 6 h. None of the anti-NET antibody deposits in a healthy kidney from untreated wild-type mice.

**Table 1:** Sequence analysis of the mAbs cloned from SLE patients.

	VH family		Number of mutations	DH	JH		Negative charge	Positive charge	CDR3	CDR3 length		VL		JL	CDR3	Negative charge	Positive charge
157 B9	V3-23*01	95.83%	12	D3-10*01	J5*02		0	2	CAKVGGIPYYGSGRWFGPW	17	λ	V3-1*01	96.77%	J2*01	CQAWDSSIHVVF	1	1
557 D7	V3-23*01	97.22%	8	D6-19*01 F	J4*02 F		1	2	CAKAPRTSGFPFDYW	13	κ	V3-20*01 F	98.43%	J4*01 F	CQQYGRSPLTF	0	1
157 B11	V3-74*01 F	98.61%	4	D6-6*01 F	J5*02 F		1	3	CARDHKYRSSGNWFDPW	15	λ	V3-1*01	97.85%	J4*01 F	CQQAWDNHSAVF	1	1
557A3	V3-23*01	98.96%	4	D3-10*	6*02 F		2	4	CAKELPYSGRWRKGMDVW	16	κ	V3-20*01 F	97.08%	J1*01 F	CQQYDSSRWTF	1	1
157 D4	V3-23*01	92.01%	23	D2-15*01	J4*01		0	2	CAKRGAAGTPPIFW	12	κ	V3-20*01	97.52%	J2*01	CQQYGSSPSTF	0	0
256 E4	V3-23*04	98.61%	4	D4-11*01	J6*02		1	1	CAKAPGSLYYYYGMDVW	15	κ	V1-46*01	98.61%	J3*02	CLLSYSGAWVF	0	0
557 F4	V3-21*01 F	98.61%	4		J4*02 F		2	2	CARERYSDYW	8	λ	V2-14*01 F	98.26%	J1*01	CSSYTSSRTRVF	0	2
157 D3	V3-7*01	97.57%	3	D3-16*02	J4*02		0	1	CARGIIVMYYW	9	κ	V3-20*01	99.29%	J1*01	CQQYGSSSRTF	0	1
557 F8	V3-48*02 F	99.65%	1 (3)	4-17*01 F	J6*02 F		2	1	CARLTTDYYYYGMDVW	16	κ	V3-20*01 F	93.80%	J1*01 F	CQQYVASPWTF	0	0
557 A2	V1-69*01	95.14%	12	D3-22	J4*02		1	3	CATMIVGAKHFDHW	12	κ	V3-11*01 F	95.38%	J2*01 F	CQHRIKWKYTF	0	4
557 E2	V2-14*01 F	96.88%	10		J2*01		0	1	CSSYRSSSTVVF	10	λ	V2-14*01 F	96.88%	J2*01	CSSYRSSSTVVF	0	1
157 B1	V3-23*04 F	99.65%	1	2-15*01 F	J4*02 F		1	3	CAKARVFCRGGSCYPLPVDYW	19	λ	V3-1*01	97.85%	J2*01	CQAWDNSAVF	1	0
557 D5	V3-23*01	100.00%	0	D6-19*01 F	J4*02 F		2	4	CAKVRGRAEVAGRFFDYW	16	λ	V2-14*01 F	96.88%	J1*01 F	CQSYGRSPLVF	0	1
156 E6	V3-20*01	98.61%	4	6-13*01	4*02		1	2	CARANLQLAHFDYW	12	κ	KV1-12*01	98.74%	J4*01	CQQANSFPLTF	0	0
156 D4	V3-30*03	86.46%	39	3-22*01	3*02		3	2	CAREMIVRALDAFDIW	14	κ	KV1-27*01	92.46%	J1*01	CQKYAGVPKTF	0	2
157 E3	V1-69*06	98.96%	4	D3-10*01	J4*02		1	2	CARDRGITMNLGYFDYW	15	κ	V3-15*01	95.57%	J5*01	CQQYNNWPPITF	0	0
157 D2	V4-38-2*01	90.62%	27	D5-12*01	J1*01		1	0	CAASGGDQIGGYTAYNPYW	17	κ	V1-39*01	91.04%	J1*01	CQQTYSVPQTF	0	0
157 A5	V1-69D*01 F	90.62%	27	D2-15*01 F	J6*03 F		1	2	CARAVVPSHINYYYYMDVW	17	κ	V2-23*02	99.31%	J2*01	CCSYVGSSTFVVF	0	0
256 A5	V3-23*04	94.79%	15	D3-10*01	J4*02		1	2	CAKVGRSLYYFDYW	14	κ	V2-14*01 F	96.88%	J1*01 F	CQSYGRSPLVF	0	1
557 F8	V3-48*02 F	99.65%	1 (3)	4-17*01 F	J6*02 F		1	1	CARLTTDYYYYGMDVW	16	κ	V3-20*01		J1*01	CQQYVASPWTF	0	0
156 E4	V7-4-1*02 F	88.19%	35	D3-22*01 F	J6*02 F		2	2	CARGGYPDFHYYGMDVW	17	κ	KV2-28*01	93.28%	KJ1*01 F	CMQTQQIWTF	0	0
157 A8	V1-2*02	95.49%	12	D3-3*01	J4*02	0	2	2	CARVAKYLAIFGDPFDYW	16	κ	V4-1*01	97.31%	J2*01	CQQYYTTPYTF	0	0
156 B7	V3-30-3*01 F	94.79%	15	3-22*01	4*02	0	3	3	CARDRSYDTIGRFFDYW	15	κ	KV4-1*01	96.93%	J4*01	CQQYYGFPLTF	0	0
156 G5	V3-74*01 F	93.40%	20	5-18*01	4*01	−1	3	2	CVRDMYGHDDYW	10	λ	V1-44*01/47	96.14%	LJ2*01	CAAWDDSLNGVVF	2	0
256 B7	V1-2*02	94.44%	15	D1-26*01	J4*02	2	1	3	CVRRSGSYYHFDYW	12	λ	V2-23*01	94.44%	J3*02	CYSYAGSSTWVF	0	0
157 D9	V1-2*02	95.49%	12	D3-3*01	J4*02	0	2	2	CARVAKYLAIFGDPFDYW	16	κ	V3-15*01	97.13%	J2*01	CQQYNNWLPYTF	0	0
557 A5	V4-59*01	95.79%	12	D6-13*01	J4*02	1	1	2	CARGGHPGYSSSWPFDYW	16	λ	V1-44*01/47	96.14%	J3*02	CAAYAGSSTWVF	0	0
157 C4	V4-38-2*02	94.79%	15	D3-3*01	J6*03	0	1	1	CARGTIFGGPMDVW	12	κ	V3-15*01	97.13%	J2*01	CQQYNNWLPYTF	0	0
157 C2	V1-18*01	95.14%	12	D2-15*01	J2*01	1	2	3	CAREGRGSWFPRYFDLW	15	λ	V2-14*01	95.14%	J2*01	CSSNISSSTVVF	0	0
557 A1	V1-69D*01 F	95.83%	13	D4-17*01 F	J4*02 F	−2	3	1	CTREDYGVEYW	11	κ	V1D-39*01 F	95.93%	J1*01 F	CQQSYSTLRAF	0	1
156 B9	V4-59*08	98.25%	5	5-18*01	4*02	2	1	3	CARHSIQVWPHYFDLW	14	κ	KV1-39*01	96.36%	J4*01	CQQSYSTPRTF	0	1
157 A2	V4-59*01 F	98.95%	3	D3-10*01 F	J3*02 F	2	1	3	CARVPRHYYGLTPYPLPNAFDIW	21	λ	V2-23*02	99.31%	J2*01	CCSYVGSSTFVVF	0	0
156 E4	V7-4-1*02 F	88.19%	35	D3-22*01 F	J6*02 F	0	2	2	CARGGYPDFHYYGMDVW	15	κ	V2D-28*01 F	93.28%	J1*01 F	CMQTQQIWTF	0	0
156 F3	V3-74*01 F	89.24%	32	3-10*02	4*01	0	2	2	CVRDMFGHLDYW	10	λ	V1-47*02/44	95.79%	LJ1*01 F	CAAWDASLSGYLF	1	0
156 E7	V3-30-3*01 F	93.06%	20	D3-3*01 F	IGHJ4*02 F	0	2	2	CAREVLTIFGVRTASSDYW	17	κ	1D-39*01 F		J2*04 F	CQQSFTTARDF	1	1
157 E9	V4-38-2*02	97.22%	3	D6-13*01	J4*02	0	1	1	CASCIAAAAHFDFW	12	λ	V2-11*01	98.61%	J3*02	CCSYAGSYTLVF	0	0
557 E5	V3-74*01 F	94.44%	9	D3-22*01 F	J4*02 F	−3	4	1	CARDPDYYDTSGLTDW	14	κ	V2-23*02		LJ1*01 F	CAAWDDSLNGYLF	2	0
157 D10	V4-38-2*02	94.79%	15	D3-3*01	J6*03	0	1	1	CARGTIFGGPMDVW	12	κ	V4-1*01	97.31%	J2*01	CQQYYTTPYTF	0	0
157 E1	V4-30-4*01 F	90.38%	28	D3-3*01 F	J1*01 F	1	0	1	CATYAFWSGSFQHW	12	κ	V1-39*01	98.26%	J2*01	YQQSYGTPMYTF	0	0
557 F10	V5-51*01 F	90.62%	27	D2-15*01 F	J5*02 F (a)	0	1	1	CARLAYCSGVACWGWFDPW	17	κ	V3-20*01 F		J1*01 F	CQQYVASPWTF	0	0
256 B5	V3-33*01	96.53%	10	D4-11*01	J6*02	0	2	2	CAREGSNYFYNYYNHGMDVW	18	λ	V2-11*01	97.22%	J1*01	CCSYGPNTYVF	0	0
156 C12	V3-74*01 F	87.85%	36	3-10*02	4*02	0	2	2	CVRDMFGPHDQW	10	λ	V1-47*01/44	93.68%	LJ1*01 F	CAAWDARLRGYLF	1	2
557 D2	V3-21*	99.31%	2	D3-16	J4*02	1	1	2	CARRGSSGYYDYW	11	λ	V1-36*01		J2*01F	CAAWDDRLRAVLF	2	2
256 C6	V3-30*02	92.36%	23	D2-21*02	J4*02	1	1	2	CARGGPPYGTHFDCW	13	κ	V1-47*02	93.68%	J2*01	CAAWDDSLSVVVV	2	0
557 D12	V3-21*	99.31%	2	D3-16	J4*02	1	1	2	CARRGSSGYYDYW	11	κ	V3-11*01 F		J5*01 F	CQQRSKWLPATF	0	2

## Discussion

Our study revealed a previously unknown subset of pathogenic anti-dsDNA antibody that enhances the inflammation in SLE. Our study also reveals a novel pathogenic mechanism in SLE, that anti-dsDNA humAbs stabilise NETs and enhance inflammation. Using single plasma cell expression cloning, we identified a subset of human monoclonal anti-dsDNA antibodies, characterised by binding to decondensed chromatin in NETs which localises to the damaged kidney in vivo and promotes the expression of IFN-α by monocytes in an FcR-dependent manner. Our results encapsulate two main features of nephritogenic antibodies; deposition within the kidney and activation of inflammatory immune responses by inhibiting NET digestion.

Our study for the first time identifies that anti-dsDNA antibodies that bind to both histones and Crithidia DNA prevent NET from nuclease digestion. Although the clinical significance of anti-dsDNA antibodies is well known, there are still significant gaps in our understanding as to how they are involved in disease progression. Analysis of murine monoclonal antibodies shows that only some anti-dsDNA antibodies cause tissue damage and lupus nephritis^5^. Level of anti-dsDNA antibodies also does not correlate with disease activity; some individuals have a high titre of anti-dsDNA antibodies and remain well^1^. These findings indicate that anti-dsDNA antibodies are not all alike and the antigen-binding specificities may determine their pathogenicity^1,28^. Histone ELISA and dsDNA ELISA were selected because DNA, histone and DNA-histone complexes are the main component of the nucleosome in vivo, and anti-chromatin/nucleosome antibodies were found to be more correlated with disease activity^29–31^. *Crithidia luciliae* test was also used for the characterisation because it has a higher specificity for SLE as it only detects anti-dsDNA antibodies while ELISA detects both anti-ssDNA and anti-dsDNA antibodies^32^.

Previous studies found that some acute SLE patients have clonal expanded autoreactive VH4-34^+^ B cells, which produce polyreactive antibodies (9G4 antibodies) that react with dsDNA, histone, chromatin and apoptotic cells^33^. The 9G4 antibodies are correlated with disease activity, and clinical manifestations but the pathogenic function of them in comparison with anti-dsDNA antibody, in general, is still unexplored^4,33^. In this study, we observed clonal expansion of VH3-23^+^ plasmablasts in group A antibodies, which are also polyreactive to dsDNA, histone and apoptotic cells. However, we did not find any VH4-34 antibodies from the two acute SLE patients in our study, which suggests that additional autoreactive VH antibody-secreting cells (ASCs) expansion may also be correlated with disease activity. We also found that group A humAbs have significantly higher CDR3 charge, which was suggested to contribute to the anti-dsDNA antibody autoreactivity^33–35^.

Retrospective analysis of autoantibody and inflammatory mediators in individuals who progress to SLE show anti-chromatin antibodies precede the simultaneous increase in IFN-α levels and detection of anti-dsDNA antibodies^1,36^. We extend these finding by showing that anti-dsDNA antibodies, which bind to decondensed chromatin within NETs and inhibit their breakdown. These NETs protective mAbs bind to histone 1 and Crithidia, which might cover the chromatin, thereby possibly prevent nuclease from accessing the cleavage sites. The group A mAbs also activate monocytes and endothelial cells and deposited in the kidneys of nephritis mice. In this study all anti-dsDNA antibodies which inhibited NETs degradation bound to the Crithidia DNA and histone 1 linker protein, which connects core nucleosome subunits and has a role in the formation of higher-order chromatin structures^37–39^. Unfoldment of compact chromatin structure in NETs may promote the formation of histone 1 antibodies resulting in stabilisation of anti-NETs antibody immune complexes and subsequent pro-inflammatory immune responses^13^. In addition, histone 1 is also a component of apoptotic chromatin constituents in glomerular basement dense electron deposits that are known to be targets of nephritogenic anti-DNA antibodies^5,10,40,41^. Previous studies have linked polyclonal anti-histone 1 with SLE disease activity^29,31^; however, our data showed that these antibodies can lead to inflammatory immune responses and endothelial cell activation.

Our study revealed that only this subset of cross-reactive anti-dsDNA antibodies could enhance inflammatory response. NETs is secreted by neutrophils by an active process called NETosis that sometimes trigger cell death^22,42^. NET is composed of chromatin and neutrophil granular proteins that is suggested to capture and neutralise pathogens^13^. However, NET was also found to promote an inflammatory response in SLE by stimulating immune cells or endothelial cells^13,43,44^. Previous studies revealed that undigested NETs promotes IFN-α secretion by plasmacytoid dendritic cells^10,12,15,18,45,46^. Monocytes, tissue macrophages and endothelial cells are believed to be the main site for immune complex and extracellular DNA clearance but do not secrete pro-inflammatory cytokines following NET elimination in healthy controls^47^. By contrast, incubation of monocyte with NETs isolated from patients with SLE can also activate cGAS and caspase 1 mediated inflammatory response, suggesting that certain forms of NETs are immunostimulatory^45,48^. In addition, the dsDNA complex in NETs was shown to induce type I IFN production from pDC in a TLR9 dependent manner, driven by the TLR adaptor MydD88 and IRF7^49^. Only the NETs dsDNA–protein complex but not naked DNA is able to activate TLR9 in pDC due to the stability of the complex. Furthermore, oxidised DNA from mitochondria is also found to activate cGAS intracellularly while the NETs associated proteins were shown to engage NLRP3 inflammasome, leading to IL-1 and IL-18 release^45,50^. Here we show that stabilisation of NETs by dsDNA antibodies also stimulates the expression of IFN-α by peripheral blood monocytes, NF-kB activity in endothelial cells, illustrating an alternative inflammatory pathway triggered by NETs. We also demonstrated that the anti-dsDNA antibody – NETs complex stimulate the monocyte and endothelial cells in an FcR-dependent manner. However, it should be noted that the structure of NETs stimulated by different stimuli would have a distinct structure in vivo, therefore our finding may only represent some of the NETs structure. Binding of IgG-containing immune complexes to Fc-receptors is a crucial step in the development of lupus nephritis. In murine models of SLE, activation of Fc-receptors on circulating white cells rather than renal resident cells is responsible for inflammatory immune responses and tissue damage^51,52^. Data from experimental models of SLE and clinical renal biopsies show a striking association between markers of FcγR mediated monocyte activation and renal inflammatory immune responses in SLE, highlighting the clinical relevance of NET induced monocyte activation^52–54^. The inflammatory immune response is likely to involve several diffident factors including immune complexes, complement activation, recruitment of inflammatory leukocytes and cytokine signalling. Our findings link anti-dsDNA/NET immune complex formation to several aspects of inflammatory changes causing tissue damage in LN and highlight contribution of other innate immune cells (neutrophils and monocytes) besides plasmacytoid dendritic cells in the pathogenesis of SLE.

We then exhibit that the potentially pathogenic anti-dsDNA mAbs may amplify the inflammation instead of initiating it. We demonstrate that the anti-dsDNA mAbs alone does not enhance inflammatory response when co-incubated with monocyte or endothelial cells. Furthermore, the anti-dsDNA antibodies are only localised to kidneys of nephritic mice and not to healthy controls, suggesting that monoclonal antibodies identified in this study are more likely to exacerbate inflammatory immune responses and tissue damage but not to initiate it^55–57^. Rising evidence suggesting that both anti-dsDNA antibodies and NET are more likely to amplify rather than directly cause tissue pathology^6,12,46,48,49,57–59^. Anti-dsDNA antibodies deposit in the kidney of lupus nephritis patients but the mechanism is still controversial^5,60,61^. The group A mAbs from our study rapidly bind to glomerulus of nephritic mice but not to the healthy controls, indicating that they may recognise antigens exposed during inflammation. Therefore, they probably recognise antigens that are exposed during kidney inflammation but not native glomerulus antigens as found by other groups^25,62,63^. During inflammation, increased number of cells undergo apoptosis or even secondary necrosis, leading to the release of intracellular contents including nucleosome. These nucleosomes may serve as the antigen target for the anti-dsDNA antibodies to deposit in the kidney^38,40,64^. Experimental data from murine models indicates that multiple SLE autoantibody can trigger LN and factors such as Ig isotype, subclass, charge avidity, murine strain and other factors can have a role in promoting disease^2,5,26,65^.

Evidence from murine models of SLE suggests that chromatin-containing microparticles are responsible for the initial loss of tolerance to nuclear constituents and can prime neutrophils for NETosis^27,66–71^. Netting neutrophils leads to endothelial activation and further release of chromatin-containing microparticles, resulting in perpetuating the cycle of inflammatory immune responses, accelerated atherosclerosis, thrombosis and tissue damage in the kidneys, skin and blood^10,12,45,72–74^. The pathogenic anti-dsDNA antibodies may then bind and stabilise the NETs, further stimulate the inflammatory response and tissue damage according to our findings. We, therefore, extend the current understanding of anti-dsDNA antibody-mediated inflammation during the course of SLE.

The study does have a number of limitations. The SLE disease scores were not based solely on LN activity but did incorporate other disease domains such as constitutional, musculoskeletal and cutaneous symptoms, however DsDNA antibodies are also associated with progression skin in SLE. It will also be important to determine the impact of nephritogenic anti-dsDNA antibodies using BLIAG2004 scoring criteria on objective measures of renal disease in prospective longitudinal studies in larger SLE cohorts. Further studies will be required to assess what component of the glomerular basement membrane do the group A anti-dsDNA antibodies bind, and the influence of IgG subclass on NETs stabilisation, and endothelial activation. As we have only identified few Group A antibodies further studies with larger numbers will be necessary to further clarify the function of the subsets of anti-dsDNA antibodies in LN. It should also be noted that the capacity of group A antibodies to bind to apoptotic blebs and its influence on the progression of LN will need further study. Our data offer an explanation, for the discordance between anti-dsDNA antibody concentration and SLE activity and suggests the potential of an alternative diagnostic test to monitor SLE activity, which will need to be studied, longitudinally in larger SLE disease cohorts with appropriate disease controls. As we have demonstrated the correlation between Crithidia staining, decondensed NET binding and active SLE, it is plausible to use Crithidia and decondensed NET binding for all the SLE patients as a biomarker for precision therapy. Treatments to accelerate NETs breakdown in SLE patients with strong decondensed NET binding could be considered in the future.

### Study approval

The study is approved by Imperial College Healthcare, Department of Surgery and Cancer under the ICHTB HTA licence: 12275, REC Wales approval: 12/WA/0196.

## Supplementary information

supplementary figure legends

Supplementary Figure 1

Supplementary Figure 2

Supplementary Figure 3

Supplementary Figure 4

Supplementary Figure 5

Supplementary Figure 6
