# Supplementary material for: Autoantibody-dependent amplification of inflammation in SLE
Source: Cell Death Dis. 2020 Sep 9;11(9):729. doi: 10.1038/s41419-020-02928-6 (PMC7481301; doi:10.1038/s41419-020-02928-6)
Supplement: Supplementary file 1 — supplementary figure legends [file 41419_2020_2928_MOESM1_ESM.docx]

**Supplementary Figure 1. NET digestion is weakly correlated with NET binding or anti-dsDNA level.**

NET digestion of the SLE patients on Y axis were compared with their

**(a)** anti-dsDNA level or

**(b)** NET binding on X axis.

**Supplementary Figure 2. Active SLE patients plasmablasts were isolated for mAb cloning.**

**(a)** Flow cytometry of CD19^+^CD27^+^CD38^+^ cells from active patient. **(b)** The clinical records of the two active SLE patients were summarised in the table. The titre of dsDNA antibody was measured with anti-dsDNA ELISA kit in International Units (IU).

**Supplementary Figure 3. Histone Western Blot and Crithidia test.**

**(a)** Four group A mAbs , one group B and one group C mAb were stained against histone mixtures. Group A mAbs recognize histone 1 at around 30 kDa while the group B and C mAbs recognize core histones (H2 or H3) at around 18 kDa.

**(b)** Crithidia test result of the negative and the positive mAb staining.

**Supplementary Figure 4. Gene features of ANA +ve humAbs**

**(a, b)** The length and charge of CDR3 region of each group of ANA +ve humAbs were compared. P < 0.01. **(c)** VL gene family usage of each group of humAbs were compared.

**Supplementary Figure 5. Group A humAbs enhance NET stability**

**(a)** Neutrophils were stimulated with calcium ionophore A23187 (which is produced during the growth of *Streptomyces chartreusensis) (*Santa Cruz Biotechnology Inc). The mAbs binding to the NET were visualized by ant—human IgG FITC. Bar = 25 μm, n=4

**(b)** NET was co-incubated either with anti-DENV mAb or group A mAb in the presence of DNaseI with 1% serum for 5 days. Fluorescent staining display the amount of NET left in the DNaseI solution after 5 days. Data is representative, n=3. Bar = 25 μm

**Supplementary Figure 6. mAb binding to NETs and apoptotic cells**

**(a**) Neutrophils were stimulated with calcium ionophore A23187 (which is produced during the growth of *Streptomyces chartreusensis) (*Santa Cruz Biotechnology Inc). The mAbs binding to the NET were visualized by ant—human IgG FITC. Bar = 25 μm n = 12 **(b)** NET binding of group A mAb at 0.1 ug/ml, 1 ug/ml and 10 ug/ml were visualized by anti-human IgG FITC. n = 5 **(c)** Representative binding of a group A mAb to apoptotic cell visualized by anti-human IgG FITC, n = 12

**Supplementary Figure 7. Group A humAbs did not enhance type I IFN when co-incubated with ApoC**

**(a)** Representative dose dependent effect of group A mAbs on type I IFN expression. 0.5*10^^6^ /ml CD14, NET and group A antibodies at various concentrations (1 μg/ml to 100 μg/ml) were co-incubated for 24 hours before type I expression were analysed by real-time PCR. N=3

**(b)** Apoptotic cells (induced by UV for 30 min) were co-incubated with 50 μg/ml mAbs from group A (557A3 and 157B9), group B (557A2 and 557F4) and group C (156B7 and 157D9) before adding to 0.5*10^^6^ CD14 /ml for 12 hours. The type I IFN expression were then analysed by real-time PCR after RNA extraction. ApoC co-incubated with CD14 alone was used as the baseline while the others were calculated as the fold change. N=3
