# Supplementary figures and images for: Autoantibody-dependent amplification of inflammation in SLE
Source: Cell Death Dis. 2020 Sep 9;11(9):729. doi: 10.1038/s41419-020-02928-6 (PMC7481301; doi:10.1038/s41419-020-02928-6)

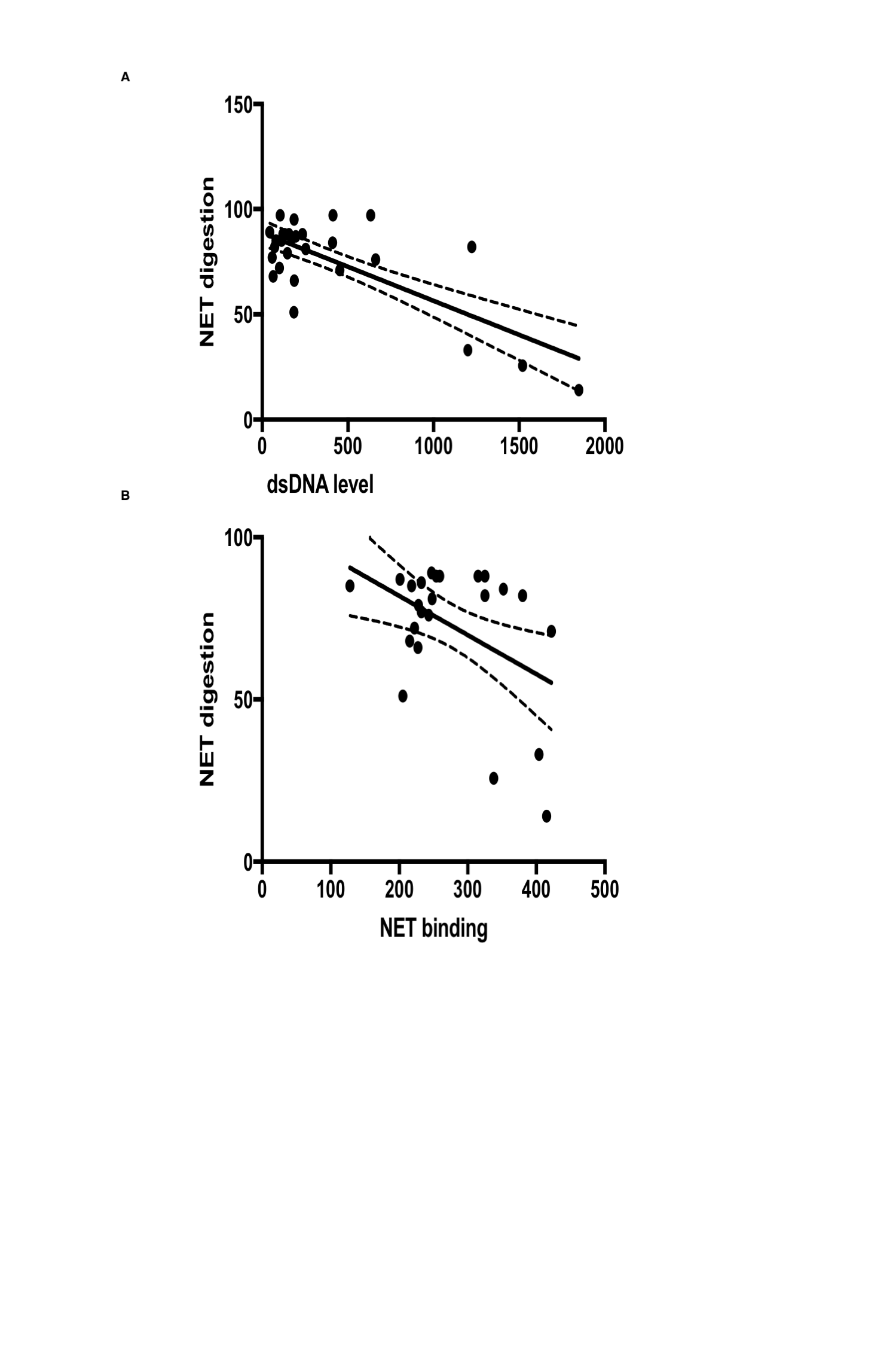

Supplement: Supplementary file 2 — Supplementary Figure 1 [file 41419_2020_2928_MOESM2_ESM.tif]

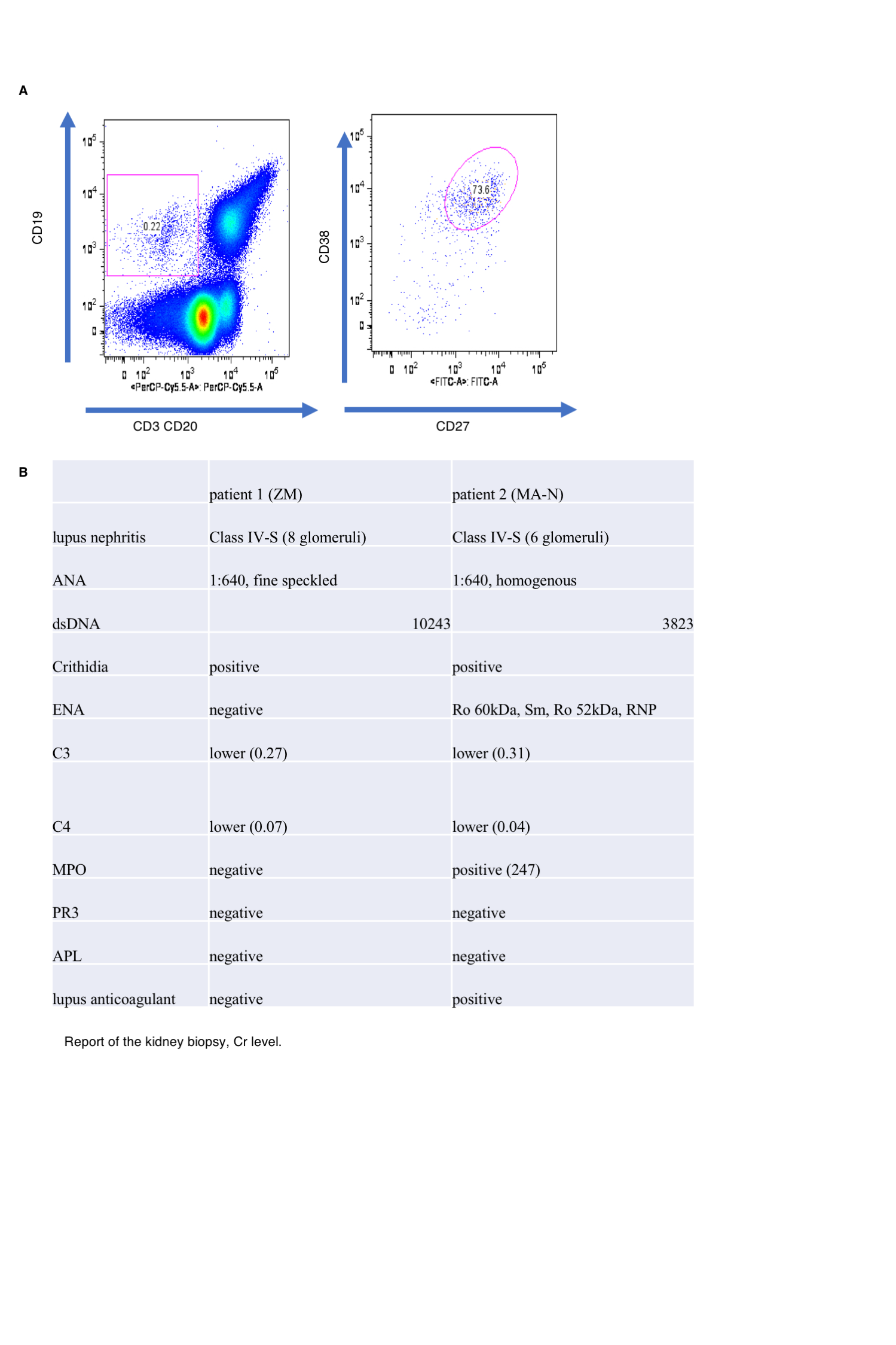

Supplement: Supplementary file 3 — Supplementary Figure 2 [file 41419_2020_2928_MOESM3_ESM.tif]

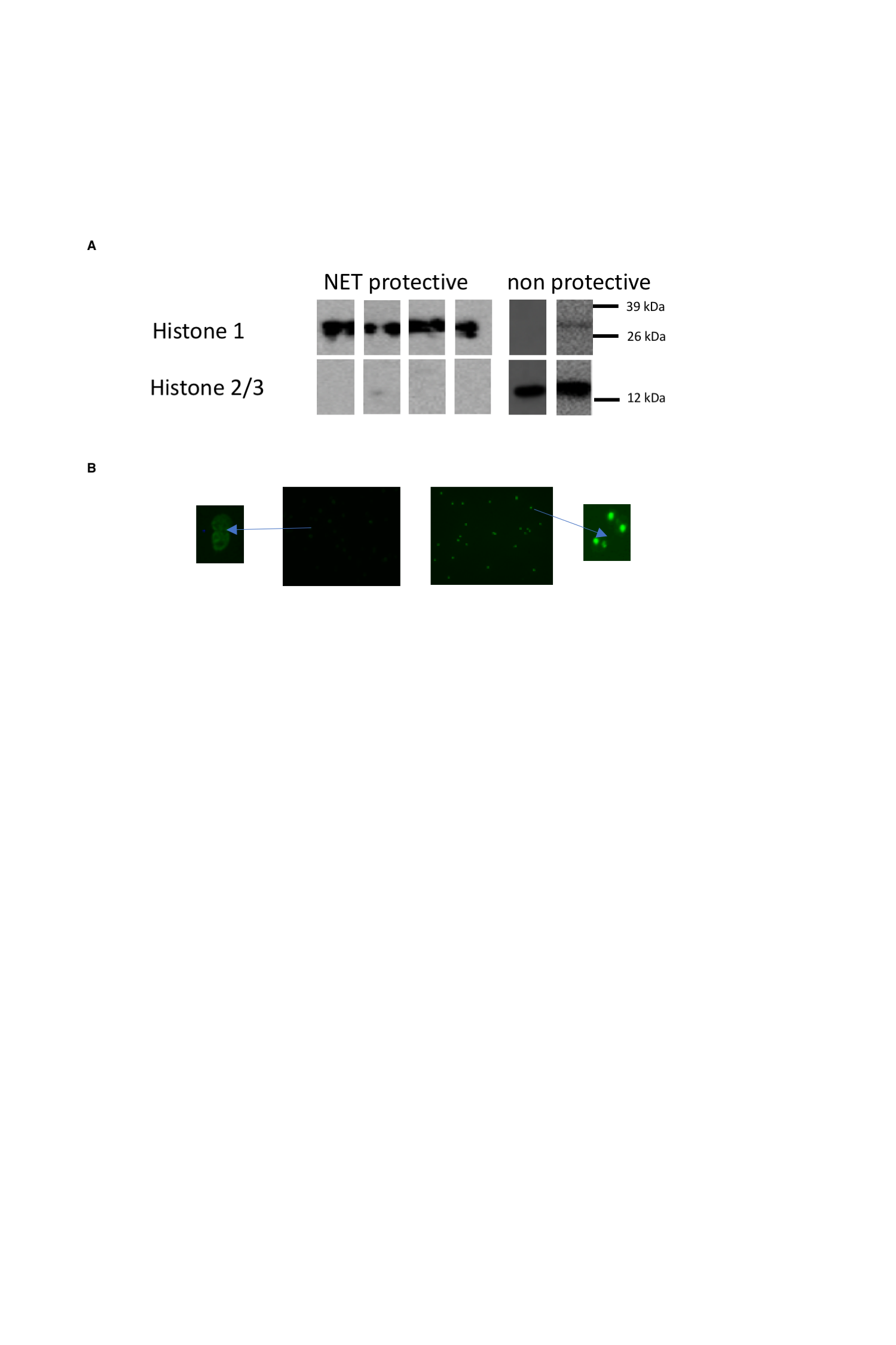

Supplement: Supplementary file 4 — Supplementary Figure 3 [file 41419_2020_2928_MOESM4_ESM.tif]

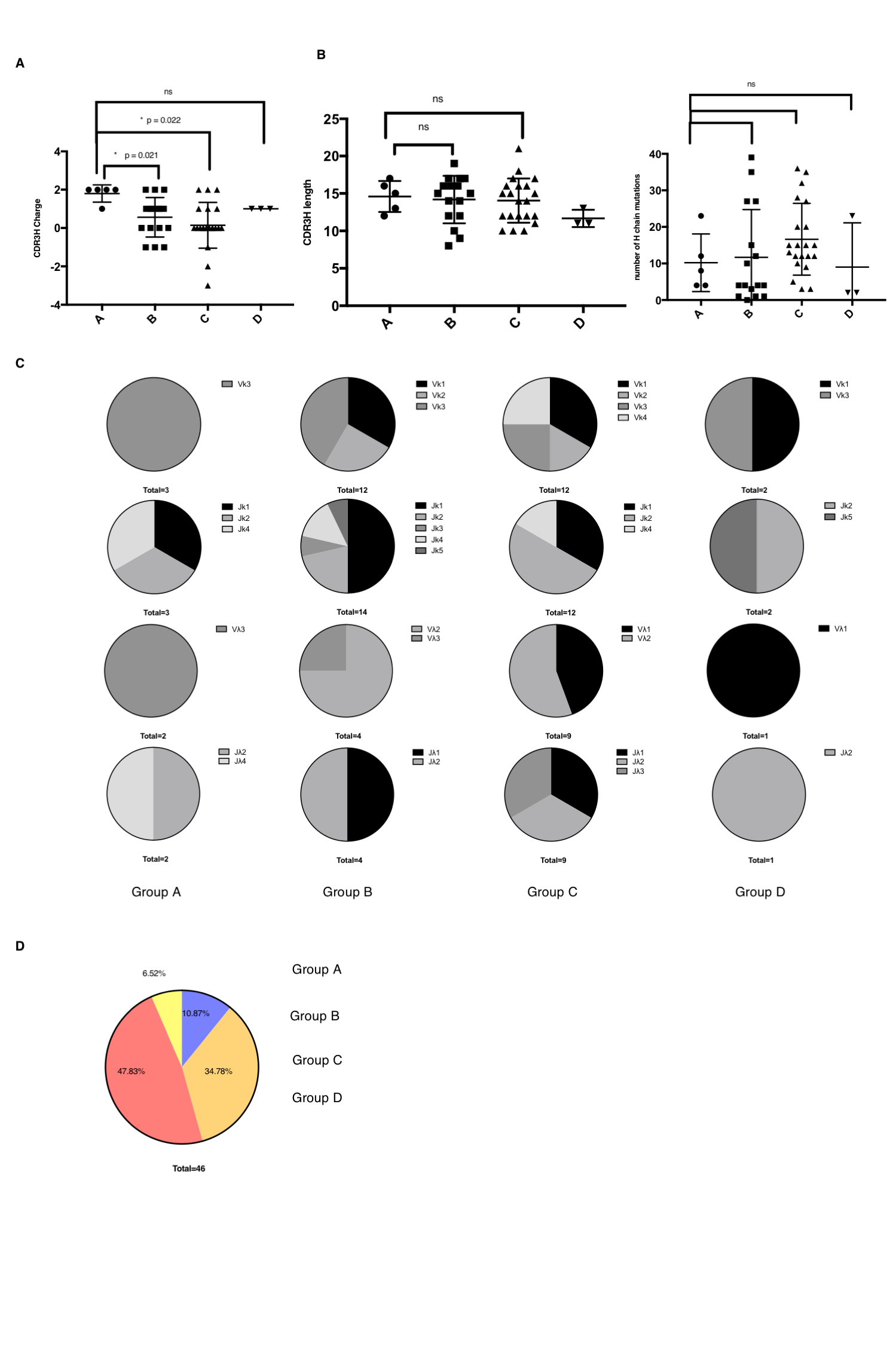

Supplement: Supplementary file 5 — Supplementary Figure 4 [file 41419_2020_2928_MOESM5_ESM.tif]

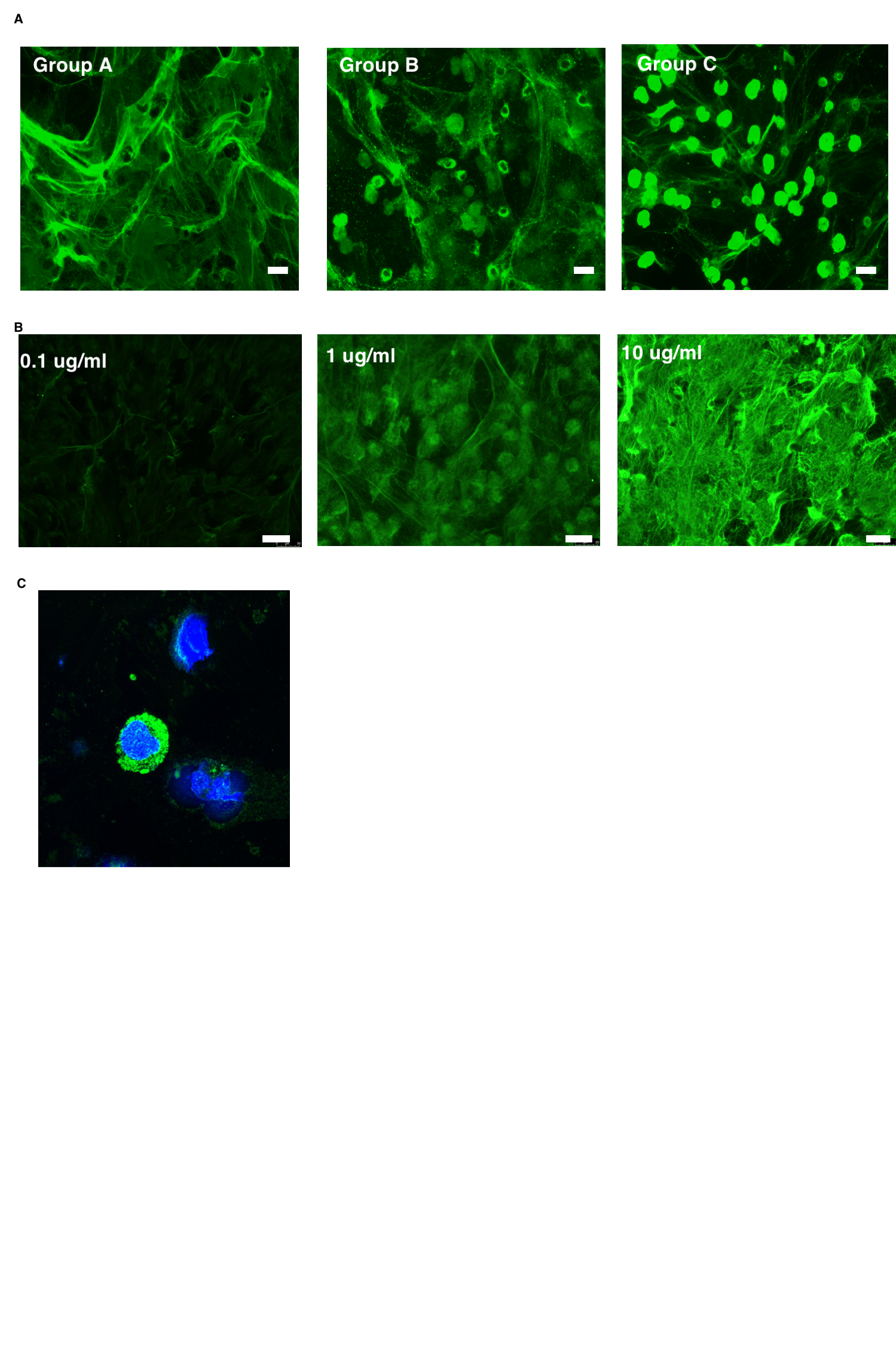

Supplement: Supplementary file 6 — Supplementary Figure 5 [file 41419_2020_2928_MOESM6_ESM.tif]

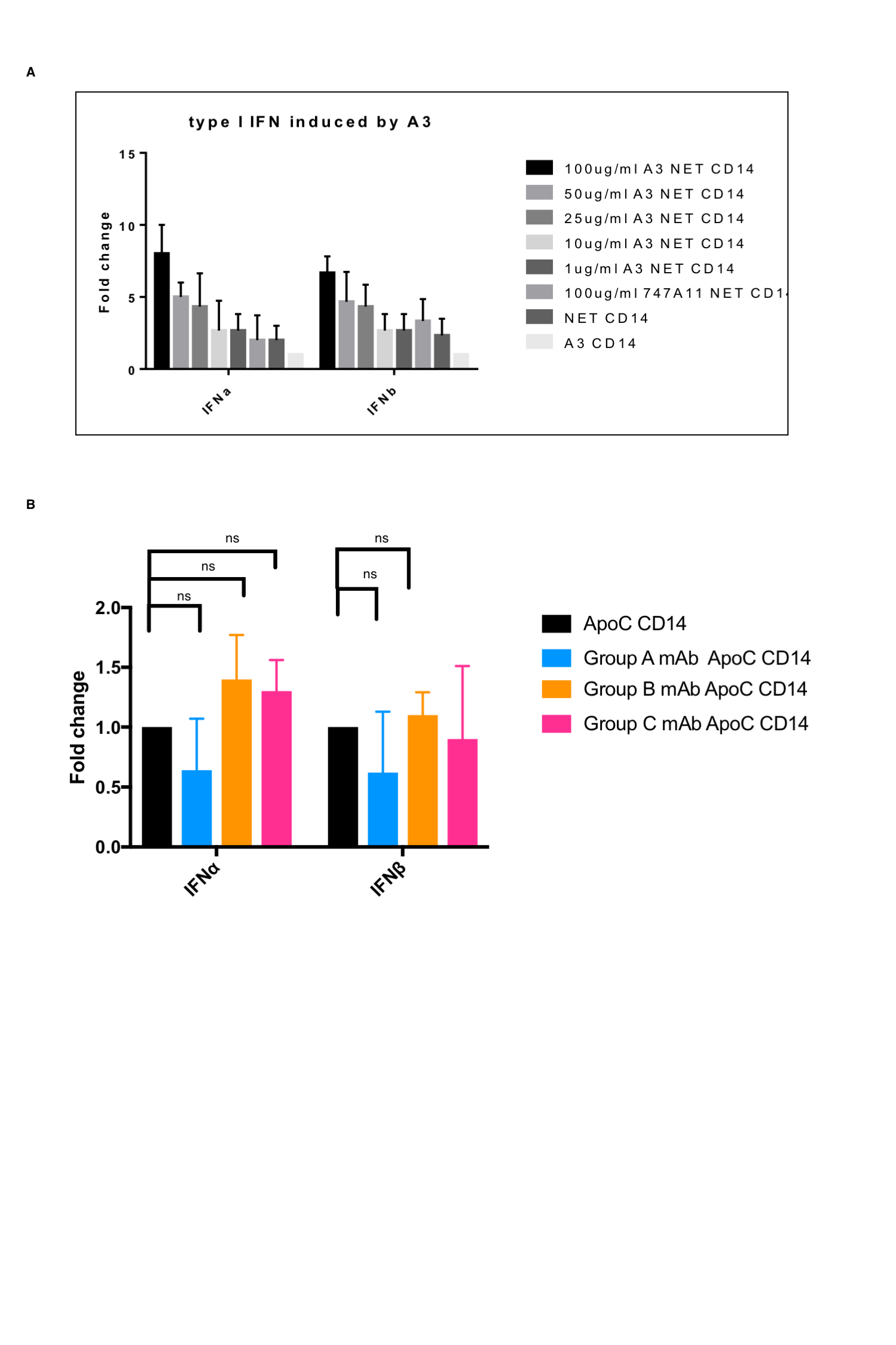

Supplement: Supplementary file 7 — Supplementary Figure 6 [file 41419_2020_2928_MOESM7_ESM.tif]
